# Analysis of early-onset bloodstream infection due to *Escherichia coli* infection in premature babies

**DOI:** 10.1097/MD.0000000000007748

**Published:** 2017-08-11

**Authors:** I-Lun Chen, Hsin-Chun Huang, Chih-Te Wu, Mei-Chen Ou-Yang, Mei-Yung Chung, Chih-Cheng Chen, Jau-Ling Suen, Chih-Hsing Hung

**Affiliations:** a Department of Pediatrics, Kaohsiung Chang Gung Memorial Hospital, Kaohsiung, Taiwan; b Department of Pediatrics, Chang Gung Memorial Hospital, Linkou, Taiwan; c Department of Pediatrics, College of Medicine, Kaohsiung Medical University, Kaohsiung, Taiwan; d Graduate Institute of Medicine, College of Medicine, Kaohsiung Medical University, Kaohsiung, Taiwan; e Department of Pediatrics, Kaohsiung Municipal Hsiao-Kang Hospital, Kaohsiung Medical University, Kaohsiung, Taiwan.

**Keywords:** *Escherichia coli*, neonate, premature, sepsis

## Abstract

In early-onset bacteremia among preterm neonates, *Escherichia coli* (*E. coli*) is the main pathogen and can cause a high mortality rate. Thus, the predictive factors of mortality and extended-spectrum β-lactamase (ESBL)-producing *E. coli* in preterm babies with *E. coli* early-onset bacteremia were reported.

We retrospectively reviewed preterm neonates who had *E. coli* bacteremia occurring within 3 days after birth between 2004 and 2015. Maternal and perinatal information were collected from their medical records and analyzed by comparing the survival and nonsurvival groups, and also the ESBL-producing and non-ESBL-producing *E. coli* bacteremia groups. Mann–Whitney *U* test, Fisher exact test, and multivariate Cox proportional-hazard model were used for statistical analysis.

A total of 27 preterm babies had *E. coli* bacteremia. The overall mortality rate was 55.56% (15 deaths). Five babies had ESBL-producing *E. coli*. The low systolic blood pressure of <48 mm Hg and low absolute neutrophil count of <2318 cells/mm^3^ were the most significant factors in predicting mortality. Moreover, the level of serum alanine aminotransferase was significantly lower in the ESBL-producing *E. coli* group than that in the non-ESBL-producing *E. coli* group.

Therefore, the lower systolic blood pressure and absolute neutrophil count were the risk factors of mortality in preterm babies with early-onset *E. coli* bacteremia, and alanine aminotransferase could be a significant factor in predicting ESBL-producing *E. coli*.

## 1 Introduction

According to the data from the World Health Organization (WHO), approximately 2.8 million deaths occurred during the neonatal period in 2013, with more than one-third (36%) resulting due to infections.^[1]^ Neonatal sepsis occurred more frequently in premature babies due to their immature immune system. In a cohort study, the incidence of early-onset neonatal sepsis (occurring within the first 72 hours of life) was 15 to 19 per 1000 live births, with Gram-negative organisms being the causative factor in more than half of these cases.^[2]^ Unfortunately, the clinical signs and symptoms of neonatal bacteremia are unspecific, and the presence of feeding intolerance, self-resolving apnea or bradycardia, mild tachypnea or tachycardia, or decreased activity may be the only warning signs before fulminant clinical deterioration. Thus, a septic work-up that includes complete cell count and differential count, C-reactive protein, urinalysis, chest x-ray, and blood, urine, or cerebrospinal fluid were arranged at the time of admission for all neonates suspected of having bacterial infection.

Neonatal bacteremia is determined on positive blood cultures,^[3]^ which may take at least 3 days. Thus, empiric antibiotics such as ampicillin and gentamicin or cefotaxime are routinely prescribed before the culture results are available for covering the common pathogens. Recently, the incidence of group B *streptococci* (GBS) infection was significantly decreased owing to the introduction of GBS intrapartum antibiotic prophylaxis, even if GBS remains a common pathogen in the early-onset bloodstream infection (BSI) in neonates.^[4]^ Moreover, *Escherichia coli* (*E. coli*) is currently the most common pathogen and has a higher mortality rate than GBS in preterm infants.^[5]^

The frequency of antimicrobial resistance such as extended-spectrum β-lactamase (ESBL)-producing *Enterobacteriaceae* is around 6% in the early-onset BSI.^[6]^ Thus, BSI with ESBL-producing *E. coli* infection in preterm babies is a big challenge to clinicians, because the routinely prescribed antibiotics do not cover ESBL-producing *E. coli*, and can result in deaths.

This study retrospectively reviewed the data from the medical records to compare the clinical characteristics and laboratory data of preterm babies with *E. coli* BSI between the survival and nonsurvival groups, and also the ESBL-producing and non-ESBL-producing groups to determine the predictive factors of *E. coli* BSI in preterm babies.

## 2 Materials and methods

This retrospective cross-sectional study reviewed all infants who were admitted to 2 medical centers located in the northern and southern Taiwan between January 2004 and July 2015. The Institutional Review Board of Kaohsiung Chang Gung Memorial Hospital approved the study protocol. The patients were enrolled in this study if *E. coli* was isolated from their blood within 3 days after birth. All were preterm neonates, gestational age of <37 weeks, without any surgically indicated diseases, or congenital anomalies. These patients were further divided into the ESBL-producing *E. coli* and the non-ESBL-producing *E. coli* groups based on their antimicrobial sensitivity tests, and also divided into survival and nonsurvival groups based on their outcome. All data were obtained from the medical records. The perinatal and maternal characteristics, including the gestational age, mode of delivery, sex, birth body weight, Apgar score, ventilator settings, neonatal antibiotics use, maternal fever, maternal antibiotics use, hours of premature rupture of membrane, initial TPR (ie, body temperature, pulse rate, and respiratory rate), and blood pressure, were compared.

Neonatal laboratory data, which were collected at the same time of blood culture, such as arterial blood gas, aspartate aminotransferase (AST), alanine aminotransferase (ALT), complete blood count, C-reactive protein, electrolytes, blood sugar, blood urea nitrogen, creatinine, prothrombin time, and activated partial thromboplastin time, were analyzed. The maternal laboratory data upon delivery, such as complete blood count, C-reactive protein, and culture of the amniotic fluid, were also reviewed.

Data were analyzed using the IBM SPSS Version 22.0 (IBM, Armonk, NY: IBM Corp) statistical software. The Mann–Whitney *U* test and Fisher exact test were used to compare the univariate analysis of continuous and binary variables of the ESBL-producing and non-ESBL-producing *E. coli* groups, and the survival and nonsurvival groups, respectively. Survival cases were defined as patients surviving during the first admission. The clinical parameters of mortality in premature infants with *E. coli* BSI were also analyzed using the multivariate Cox proportional-hazard model, which were adjusted for gestational age. Receiver-operating characteristic (ROC) statistics was applied and the areas under curve were compared to calculate their cut-off values. All data in tables were presented with the mean ± standard deviation. For all tests, statistical significance was set at *P* ≤ .05.

## 3 Results

Between January 2004 and July 2015, 27 preterm babies were diagnosed as early-onset BSI, with *E. coli* based on the isolation of these bacteria from blood culture. Among these babies, 2 also had *E. coli* pneumonia, 3 had *E. coli* urinary tract infection, 1 had *E. coli* meningitis, and 1 had bowel perforation with *E. coli* ascites. Their mean gestational age was 31.2 weeks. The mean days of hospitalization was 9.19 ± 10.21 days. Immediately after birth, all patients were treated with empiric antibiotics of ampicillin and either gentamicin or cefotaxime. The overall mortality rate was 55.56% (15 deaths). Fourteen preterm babies died within 72 hours after birth due to septic shock and cardiopulmonary failure, and 1 baby died on 19th day after birth. According to the antibiotic susceptibility test, 5 neonates had BSI caused by ESBL-producing *E. coli*. Table 1 shows the characteristics of these 5 neonates.

**Table 1:**
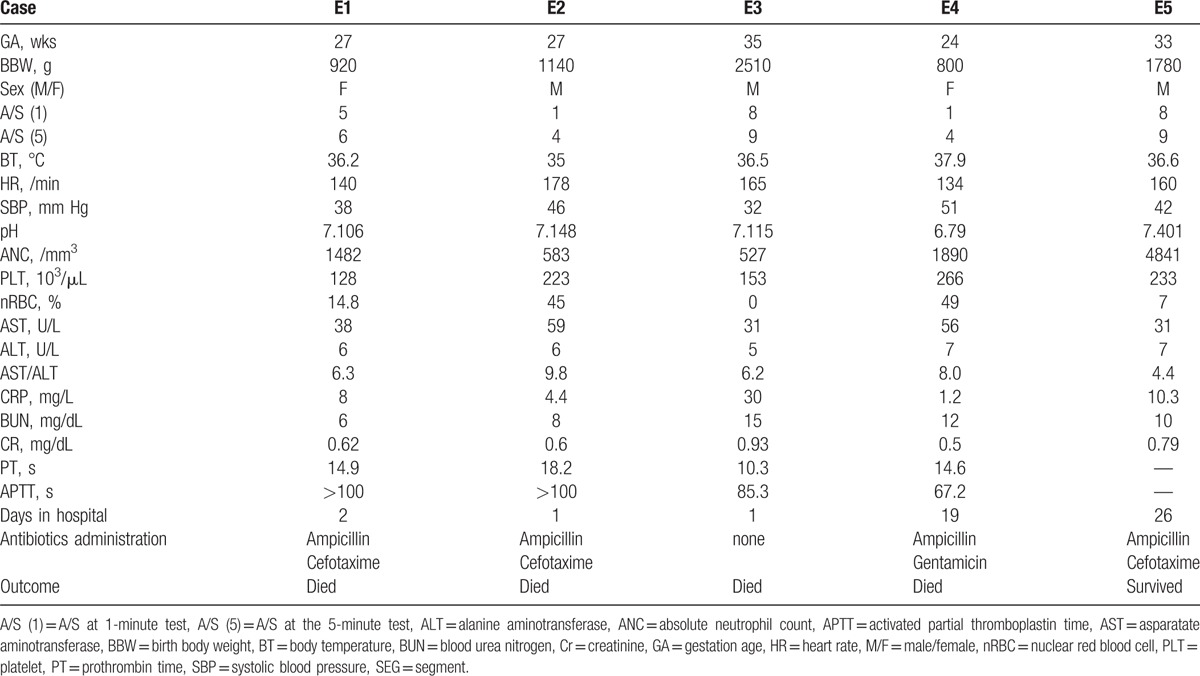
The characteristics of ESBL-producing *Escherichia coli* cases.

Comparing the perinatal conditions, neonatal outcomes, and maternal status between the ESBL-producing *E. coli* group (ESBL group) and the non-ESBL-producing *E. coli* group (non-ESBL group) (Tables 2 and 3), only the ALT was significantly lower in ESBL group than in the non-ESBL group. The mortality rates of ESBL and non-ESBL groups were 80% and 54.5%, respectively. Although the mortality rate was not significantly different from the 2 groups, that of the ESBL group was higher.

**Table 2:**
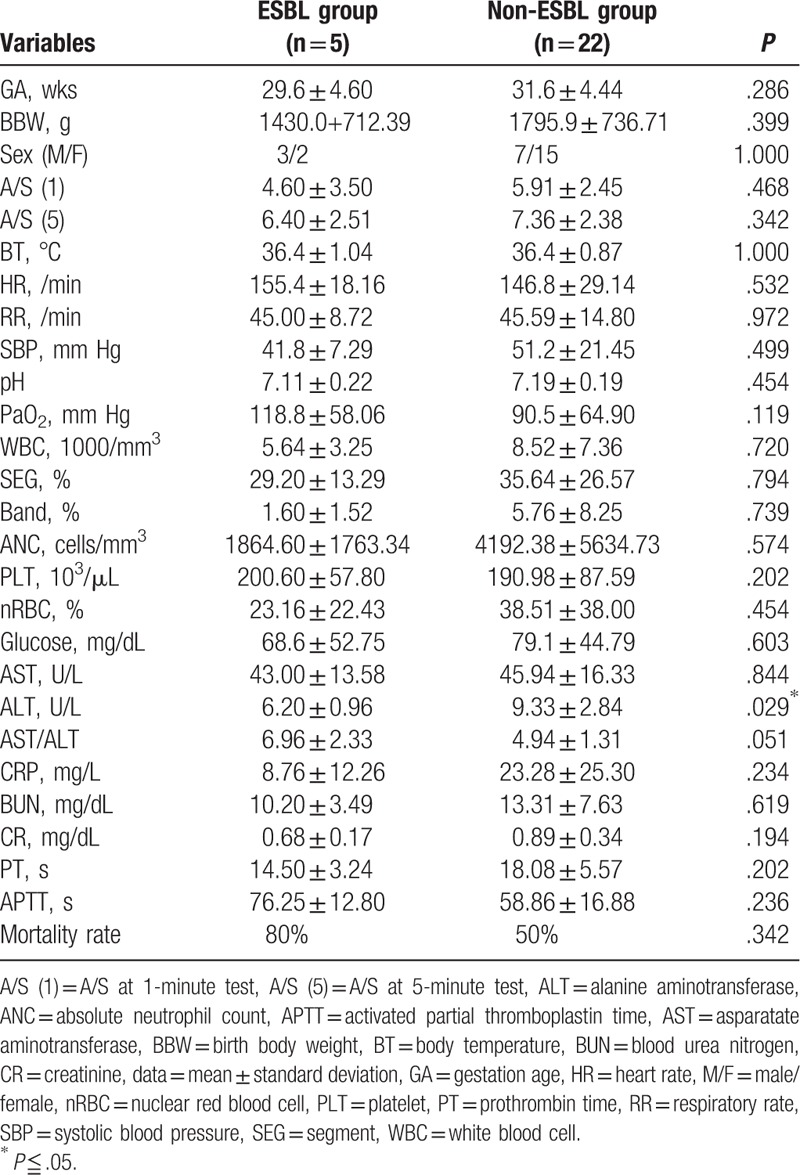
The perinatal conditions and neonatal outcome comparing ESBL-producing *E. coli* group (ESBL group) and non-ESBL-producing *E. coli* group (non-ESBL group).

**Table 3:**
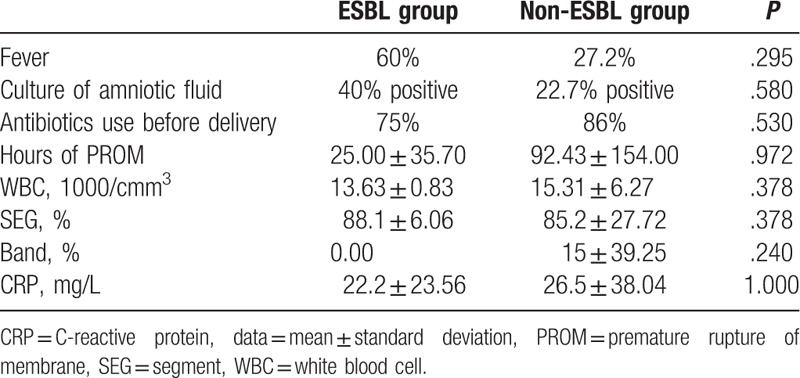
The maternal conditions comparing the ESBL-producing *E. coli* group (ESBL group) and non-ESBL-producing *E. coli* group (non-ESBL group).

The Mann–Whitney *U* test was used to determine the risk factors of mortality of the survivors and nonsurvivors. Blood acidosis, anemia, and low systolic blood pressure, gestational age, birth body weight, Apgar score, absolute neutrophil count (ANC), count of platelet count, and ALT were risk factors of mortality in preterm babies with *E. coli* BSI (Table 4). After adjusting the gestational age, low systolic blood pressure and ANC were the significant risk factors of mortality based on the multivariate Cox regression (Table 4).

**Table 4:**
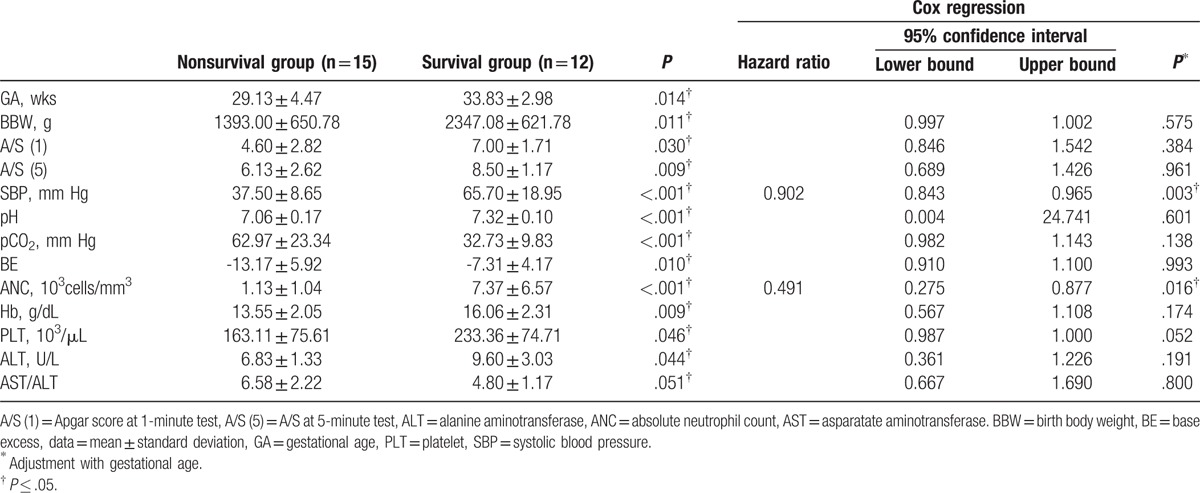
The risk factors of mortality in premature infants with *E. coli* bloodstream infection.

The predictive values of the systolic blood pressure and ANC associated with mortality were analyzed using the ROC statistics. Systolic blood pressure and ANC had a total area under the ROC curve (AUC) of 0.937 and 0.968, respectively. Their cut-off values were 48 mm Hg (77.8% sensitivity, 92.9% specificity) and 2318 cells/mm^3^ (100% sensitivity, 85.7% specificity), respectively.

## 4 Discussion

To date, this is the first study to compare the ESBL-producing *E. coli* and non-ESBL-producing *E. coli* BSI in preterm neonates. The results demonstrated that lower ALT level might be a predictive factor for ESBL-producing *E. coli* BSI. Moreover, systolic blood pressure of <48 mm Hg and neutropenia of <2318 cells/mm^3^ might be the most significant risk factors of mortality in these cases.

The liver is an important organ in response to sepsis as it produces proinflammatory cytokines and acute-phase proteins.^[7]^ Like the spleen, the liver is also the first defense against the pathogens in the blood. The interaction of Kupffer and natural killer T cells in the liver can induce an important intravascular immune response.^[8]^ A previous in vitro study revealed that cells infected with ESBL-producing or non-ESBL-producing *E. coli* could induce different host–response mechanisms and elicit different levels of interleukin (IL)-6 and IL-8.^[9]^ The ability of ESBL-producing *E. coli* to stimulate or evoke the production of reactive oxygen species (ROS) from polymorphonuclear neutrophil cells is higher than that of non-ESBL-producing *E. coli*.^[9]^ In an animal study, ROS and reactive nitrogen species were reported to cause hepatic microvascular dysfunction during sepsis.^[10]^

This study demonstrated that the ratio of serum AST and ALT in the ESBL group was almost significantly higher than that in the non-ESBL group (*P* = .051). The increased ratio of AST and ALT in infants with liver disease puts them at risk of poor clinical outcome.^[11]^ This suggested that the ESBL group might have more severe liver damage and poorer prognosis than the non-ESBL group. Moreover, serum ALT in the ESBL group was lower than that in the non-ESBL group.

Lower ALT concentration can occur in the setting of vitamin B6 deficiency.^[10]^ Vitamin B6 is an antioxidant and is a strong quencher of ROS. In the ESBL group, vitamin B6 may be consumed largely due to high ROS, resulting in decreased ALT levels.

The overall mortality rate of early-onset neonatal sepsis in premature babies was known to be as high as 34%.^[12]^ Neonates with multidrug-resistant (MDR) Gram-negative bacteremia had higher rates of fatality and infectious complications than those with non-MDR strain.^[13]^ However, a large cohort study revealed that the association of ampicillin-resistant *E. coli* BSI and mortality was not significant, and effective empiric agents could not decrease the mortality. ^[14]^ The population of this study included full-term babies, and early and late-onset BSI were analyzed together. Our study was focused on early-onset *E. coli* BSI on premature babies and revealed that ESBL group had higher mortality rate (80%) than non-ESBL group (54.5%). More than 90% of the babies in our study died within 72 hours after birth; clinicians usually knew the result of the blood culture after these babies died.

Infectious complications included any abscess formation, neurologic complications, acute renal failure, and intracardiac vegetation. BSI with septic shock could be an independent risk factor for infectious complications, which finally lead to mortality.^[15]^ In our study, the systolic blood pressure of <48 mm Hg and ANC of <2318 cells/mm^3^ might be the most significant risk factors of mortality. Septic shock and subsequent disseminated intravascular coagulation could cause intraventricular hemorrhage leading to poor outcomes. Although platelet count, prothrombin time, and activated partial thromboplastin time were not associated with mortality in our study, a previous study revealed that lower level of plasma fibrinogen was associated to death in neonatal sepsis. ^[16]^ Correct neutrophil counts provide the first line of innate immune system defense to pathogens through phagocytosis. The ANC of <1500 cells/mm^3^ is considered neutropenia, which could be congenital or acquired. In premature infants, neutropenia is a common finding, particularly in extremely low-birth-weight babies, which might be associated with an increased incidence of early-onset sepsis.^[17]^ Neutrophilia and neutropenia could happen during neonatal sepsis based on the bone marrow storage pool or the infectious pathogens. Neutropenia was usually found during sepsis in sick premature infants who had Gram-negative bacterial infection.^[18]^

Bloodstream infection is 1 of the most common causes of morbidity and mortality in preterm babies.^[19]^ Preterm neonates are prone to infections due to their immature immune function, and thus, starting antibiotic treatment immediately after birth is recommended. Nevertheless, empiric antibiotic treatment, even intravenous immunoglobulin (IVIG) or IgM-enriched IVIG^[20]^, may not prevent disease progression and mortality. A study had revealed that preterm infants who survived from severe infections were associated with white matter injury and neurodevelopmental sequelae.^[21]^ Thus, early diagnosis and timely intervention are needed to reduce disease morbidity. Previous studies had identified indicators for the early detection of neonatal sepsis. These included the ratio of abnormal immature and mature neutrophils in the cord blood ^[19]^and 16S rRNA gene sequencing in neonatal peripheral blood.^[22]^ However, phlebotomy examination of premature babies were difficult to perform because of their small blood volume and vessels. Therefore, other bio-fluid, such as urine, was reported to detect neonatal sepsis through an elevated IL-6.^[23]^

This study has shown that lower ALT was correlated with ESBL *E. coli* bacteremia in preterm neonates. Early shifting of antibiotics from empirical to an appropriate one may be needed to control the infection. Furthermore, clinicians should be alert in cases with low systolic blood pressure and neutropenia as these may lead to mortality. Early and aggressive management for these babies are warranted.

One of the limitations of this retrospective study was the information bias because of incomplete profiles as some of the babies were transferred from local hospitals after birth, so some data with regard to maternal conditions were missing. However, these mothers’ data were recalled by looking up their medical charts, and only 6 babies were admitted to our hospital via transportation system. Furthermore, owing to the small number of the ESBL group cases, we were unable to recommend directly on the association with low ALT and ESBL group. Further prospective studies with larger cohorts are needed to clarify the difference between ESBL *E. coli* and non-ESBL *E. coli* in premature babies with BSI, and determine the method to early detect ESBL *E. coli* using other bio-fluids instead of blood.

## Acknowledgment

We appreciated the Biostatistics Center, Kaohsiung Chang Gung Memorial Hospital, for statistical work.
